# Newborn screening for G6PD deficiency in HeFei, FuYang and AnQing, China: Prevalence, cut-off value, variant spectrum

**DOI:** 10.5937/jomb0-43078

**Published:** 2024-01-25

**Authors:** Hui Li, Yah Ch'ih, Meiling Li, Yulei Luo, Hao Liu, Junyang Xu, Wangsheng Song, Qingqing Ma, Ziyu Shao

**Affiliations:** 1 HeFei Women and Children Medical Care Center, HeFei City, Anhui Province, China; 2 Zhejiang Biosan Biochemical Technologies Co., Ltd, Hangzhou City, Zhejiang Province, China; 3 FuYang Maternal and Child Health Family Planning Service Center, FuYang City, Anhui Province, China; 4 AnQing Maternal and Child Health Family Planning Service Center, AnQing City, Anhui Province, China

**Keywords:** G6PD deficiency, newborn screening, ROC curve, variant spectrum, prevalence, nedostatak G6PD, skrining novorođenčadi, ROC kriva, varijantni spektar, prevalencija

## Abstract

**Background:**

Glucose-6-phosphate dehydrogenase (G6PD) deficiency is an X-linked recessive Mendelian genetic disorder characterized by neonatal jaundice and hemolytic anemia, affecting more than 400 million people worldwide. The purpose of this research was to investigate prevalence rates of G6PD deficiency and to evaluate and establish specific cut-off values in early prediction of G6PD deficiency by regions (HeFei, FuYang, AnQing) on different seasons, as well as to investigate the frequencies of G6PD gene mutations among three regions mentioned above.

**Methods:**

A total of 31,482 neonates (21,402, 7680, and 2340 for HeFei, FuYang, and AnQing cities, respectively) were recruited. Positive subjects were recalled to attend genetic tests for diagnosis. G6PD activity on the Genetic screening processor (GSP analyzer, 2021-0010) was measured following the manufactureržs protocol. The cut-off value was first set to 35 U/dL. The receiver operating characteristics (ROC) curve was employed to assess and compare the efficiency in predicting G6PD deficiency among HeFei, FuYang, and AnQing cities in different seasons.

## Abstract (sr)

**Uvod:**

Nedostatak glukoza-6-fosfat dehidrogenaze (G6PD) je recesivni Mendelov genetski poremećaj povezan sa X hromozomom koji karakteriše neonatalna žutica i hemolitička anemija, koji pogađa više od 400 miliona ljudi širom sveta. Svrha ovog istraživanja je bila da se istraže stope prevalencije nedostatka G6PD i da se procene i utvrde specifične granične vrednosti u ranom predviđanju nedostatka G6PD po regionima (HeFei, FuIang, AnKing) u različitim godišnjim dobima, kao i da se ispita učestalost G6PD genske mutacije u okviru tri gore pomenuta regiona.

**Metode:**

Ukupno je uključeno 31.482 novorođenčadi (21.402, 7.680 i 2.340 za gradova HeFei, FuYang i AnQing). Pozitivni subjekti su pozvani da prisustvuju genetskim testovima radi postavljanja dijagnoze. Aktivnost G6PD na procesoru genetskog skrininga (GSP analizator, 2021-0010) je merena prema protokolu proizvođača. Granična vrednost je prvo postavljena na 35 U/dL. Kriva radnih karakteristika prijemnika (ROC) je korišćena da se proceni i uporedi efikasnost u predviđanju nedostatka G6PD u gradovima HeFei, FuYang i AnQing u različitim godišnjim dobima.

**Rezultati:**

Na nedostatak G6PD je testirano 31,482 novorođenčadi, a 29 novorođenčadi je identifikovano sa nedo statkom G6PD, sa stopom prevalencije od 0,09%. Stopa incidencije nedostatka G6PD je bila 0,10% (22/21.380) za HeFei, 0,04% (3/7.667) za FuYang i 0,17% (4/2.396) za AnQing. Optimalne granične vrednosti ROC krive u predviđanju nedostatka G6PD su bile 26,55 U/dL za HeFei (proleće: 21,80 U/dL, leto: 26,55 U/dL, zima: 23,16 U/dL), 6,35 U/dL za grad FuYang, 7,15 U/dL za AnQing. R463H (17/57, 29,82%) je bila najčešća varijacija, zatim R459L (15/57, 26,31%), H32R (8/57, 14,04%), L342F (6/57, 10,53%), V291M (6/57, 10,53%).

**Zaključak:**

Ustanovili smo optimalne granične vrednosti G6PD aktivnosti u predviđanju nedostatka G6PD u gradovima HeFei, FuYang i AnQing u različitim godišnjim dobima. Ovo će pomoći u ranom otkrivanju nedostatka G6PD kod novorođenčadi u gore pomenutim regionima. Takođe smo istražili prevalence po regionima i otkrili mutacije frekvencije. Ovi rezultati će pomoći u promociji G6PD skrininga i postavljanju precizne dijagnoze za provinciju Anhui.

## Introduction

Glucose-6-phosphate dehydrogenase (G6PD) deficiency, known as favism, is an X-linked recessive Mendelian genetic disorder [1], which is characterized by neonatal jaundice and hemolytic anemia [2] when triggered by oxidative drugs, infection and the intake of fava beans [3]. Mutations in the G6PD gene can result in reduced activity and stability of the G6PD enzyme, which is the crucial enzyme of the pentose phosphate pathway that produces reduced nicotinamide adenine dinucleotide phosphate (NADPH) to maintain sufficient levels of reduced glutathione [4] [5]. Red blood cells would lyse when exposed to the factors mentioned above if there was an insufficient supply of NADPH and glutathione, which is the reason for the aforementioned clinical symptoms [6]. G6PD deficiency affects more than 400 million people worldwide [5] [7] [8], especially prevalent in malaria-endemic areas [9], such as Africa, Oceania, Asia, and Mediterranean Europe [9] [10]. G6PD deficiency is also common in China [11] [12]. Nevertheless, the prevalence of G6PD deficiency, related to specific geographic regions and associated with ethnicities, is highly variable around China [13]. The highest incidence of G6PD deficiency has been reported in southern China [14] [15] [16], for instance, in Guangdong, Yunnan, and Guangxi provinces. However, we have not yet known the precise incidence of G6PD deficiency in Anhui Province.

There is no cure for G6PD deficiency, as is true for many genetic disorders, and most G6PD deficiency individuals are asymptomatic. Hence, performing a G6PD deficiency screening program effectively facilitates early diagnosis and timely intervention. G6PD enzyme activity tests were performed within Newborn Screening programs in most provinces of China [17]. According to the largest Youden Index, a specific optimal cut-off value generated by receiver operating characteristic (ROC) curve analysis was often to be used to predict G6PD deficiency and evaluate for diagnostic accuracy based on sensitivity, specificity, and the area under the ROC curve (AUC), having to discriminate all patients with G6PD levels accurately below a preset cut-off value, which is often followed with manufacturer's protocol. It is widely known that enzymatic activity may be affected by seasons and temperatures in vitro and, consequently, possess a different level of G6PD activity in different parts and seasons. Furthermore, through random Xchromosome inactivation, females heterozygous for G6PD could manifest various degrees of G6PD deficiency [18], which was called intermediate G6PD activities between typical normal and deficient G6PD activities [19]. Therefore, the cut-off values for newborn screening of G6PD deficiency should optimize based on geographical regions, seasons, and gender.

Cut-off values of G6PD activity to determine G6PD deficiency differ for genetic background, for the G6PD gene is highly polymorphic with many variants, which could lead to decreased G6PD activity in erythrocytes or G6PD deficiency [20].To date, 270 mutations of the G6PD gene have been identified as pathogenic or likely pathogenic (https://www.ncbi.nlm.nih.gov/clinvar/?term=g6pd%5Bgene%5D&redir=gene), and over 200 pathogenic variants have been reported in China [21]. As a previous study reported [21], G6PD gene variants K459M, L463H, A32G, P342S, and Q291K account for approximately 95% of the causative reasons for G6PD deficiency individuals in the Chinese population. However, the distribution of G6PD gene variants is related to geographical regions and ethnic groups, as reported. Hence, it is believed to be important to discover characteristics of the native G6PD variant spectrum.

In this study, we first established G6PD activity cut-off values of HeFei, FuYang, and AnQing because of geographical regions and seasons, based on a population cohort of 31482 neonates participating in Newborn Screening between December 2020 and October 2021. We also profiled the characteristics of the G6PD variant spectrum of the areas mentioned above and revealed the prevalence of the regions.

## Materials and methods

### Subjects

A total of 31482 neonates (21402, 7680, and 2340 for HeFei, FuYang, and AnQing, respectively) were recruited through the Newborn Screening program at the newborn screening centre of HeFei Women and Children Medical Care Centre, FuYang Maternal and Child Health Family Planning Service Center, and AnQing Maternal and Child Health Family Planning Service Center between December 2020 and October 2021. Details are available in Table 1. Dried blood spots (DBS) were collected from a heel stick within 48 hours after birth. The guardians of all subjects in the study signed informed consent, which was approved by the Medical Ethics Committee of the hospital mentioned above.

**Table 1:** Characteristics of newborns screened by G6PD newborn screening. Normal: Neonates who were not diagnosed with G6PD deficiency by Newborn Screening.<br>Normal: Nenoates, diagnosed with G6PD deficiency by Newborn Screening.<br>H: Kruskal-Wallis Test; a: indicating that it did not fit with a Gaussian distribution after being assessed by the KS test.

	Normal N = 31453	Patients N = 29	H	P
Age at initial testing^a^	4.00 (3.00~5.00)	4.00 (4.00~7.00)	8.809*	0.033
Gender				
Male	11405	22	9.995*	0.002
Female	9974	0		
No record	10074	7		
Gestational age^a^ (weeks)	39.29 (38.57~40.14)	39.71 (37.00~39.93)	4.984*	0.026
Birth Weight (g)^a^	3350 (3050~3650)	3200 (2850~3530)	2.805	0.094
Region				
HF	21380	22	0.365	0.546
FY	7677	3		
AnQ	2396	4		

### Determination of G6PD enzyme activity

G6PD enzyme activity was determined using a Genetic screening processor (GSP analyzer, 2021-0010), Panthera-PuncherTM 9 blood spot punching system, and Nenatal G6PD Kit, which were all purchased from Perkin Elmer (Perkin Elmer, Waltham, Massachusetts, United States), according to the manufacturer's instructions. In brief, G6PD calibrators, G6PD controls, and DBS samples were added separately to 96-well microplates, and the detection was performed using a GSP analyzer. According to the manufacturer's recommendation, the cut-off values of G6PD activity for G6PD deficiency were set to 35 U/dL. Subjects with G6P-D activity less than 35 U/dL were assayed using another DBS again.

### Diagnosis test for G6PD deficiency

Subjects whose G6PD activity was less than 35 U/dL after the second test were defined as having positive results and recalled to attend a genetic test. Venous blood was gathered from each positive subject and then sent to the Clinical Laboratory of Zhejiang Biosan Biochemical Technologies Co., Ltd for laboratory tests. A client was diagnosed with G6PD deficiency positive if the genetic test results were positive.

A genetic test examined the 12 common hotspot mutation sites of the G6PD gene in the Chinese population, accounting for more than 95% of G6PD deficiency [21]. Twelve common G6PD variants were: c.1360C>T(R454C), c.1376G>T(R459L), c.1388G >A(R463H), c.871G>A(V291M), c.1004C> A(A335D), c.1024C>T(L342F), c.95A>G(H32R), c.383T>C(L128P), c.392G>T(G131V), c.487G> A(G163S), c.592C>T(R198C), c.517T>C(F173L). The reaction was performed on a SLAN-96S real-time PCR system (Hongshi, Shanghai, China). Supplementary Table 1 outlines PCR amplification and melting curve conditions in detail. By plotting the negative derivative of fluorescence versus temperature (dF/dT) as a function of temperature, Tm values and melting curves were generated automatically, being carried out with the software SLAN 8.0 (Hongshi).

### Statical Analysis

Statical Analysis was performed using the SPSS analysis software(SPSS version 25.0 for Windows, SPSS Inc. Chicago, IL, United States). Non-normally distributed data was conducted on the Mann-Whitney U test and expressed as quartiles P50 (P25~P75). P < 0.05 was considered statistically significant. Receiver operative characteristics (ROC) curves were performed in MedCalc (MedCalc Soft-ware v.19.05, Mariakerke, Belgium). Mapping was made using GraphPad Prism 9.00 (GraphPad Software, La Jolla, CA, USA).

## Results

### A review of Newborn Screening and diagnosis of glucose-6 phosphate dehydrogenase deficiency

As depicted in Table 1, 31482 participants were screened for G6PD deficiency, including 29 (0.09%) neonates finally identified with G6PD deficiency. Among the 29 neonates, 22 were males, and seven were not documented, of whom 22 (0.10%) were from HeFei, 3 (0.04%) were from FuYang, and 4 (0.17%) were from AnQing. Several characteristics differed significantly across age at initial testing, gender, and gestational age between the two groups, but had no significant differences in Birth Weight and Region. The normal group (Newborns without G6PD deficiency) showed smaller values in age at initial testing and Gestational age. Neonates confirmed G6PD deficiency were predominantly males (p = 0.002 < 0.05).

### Effects of geographic regions and season on G6PD activity

**Figure 1:**
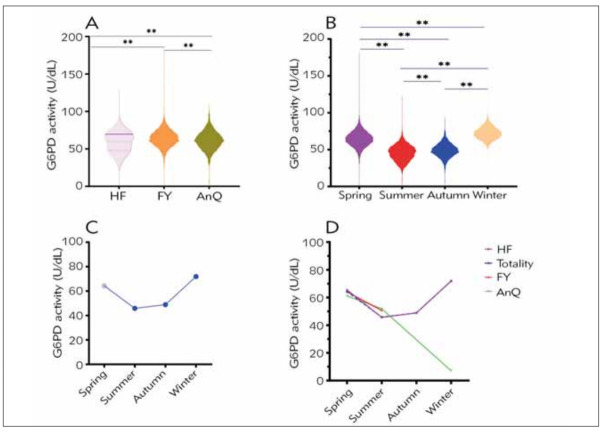
Effects of geographic regions and season on G6PD activity. ** represents p < 0.01 Abbreviations: HF, HeFei city, FY, FuYang city, AnQ for AnQing.<br>1A Comparison of enzymatic activity of HF, FY, and AnQ.<br>1B Comparison of spring, summer, autumn, and winter enzymatic activity.<br>1C Median of G6PD activity changes from Seasons.<br>1D G6PD activity distributions by regions (HeFei, FuYang, AnQing) in different seasons.

We compared G6PD activity between geographical regions and seasons. In general, the distribution medians were significantly different (p<0.001) (59.58 (range 39.76-69.10)), (63.90 (range 57.00–71.00)) and (63.14 (range 53.29–69.05)) between HeFei, FuYang, and AnQing, respectively, as presented in Figure 1A. As shown in Figure 1B and Figure 1C, significant seasonal differences existed in G6PD activity. G6PD activity presented declining trends during spring and summer and increasing trends from summer to winter (Figure 1C). In contrast, G6PD activity was significantly different between 4 categories: median values for spring, summer, autumn, and winter were 64.3000 (range 57.80–70.91), 45.83 (range 38.14–52.74), 48.89 (range 43.37–54.74), 71.8900 (range 66.43–77.65), respectively (Table 1). Due to no participants engaged in screening in certain seasons (FuYang in autumn, AnQing in autumn and winter), we could only display the whole season G6PD activity of HeFei, which was consistent with the tendency of Totality (Figure 1D).

### Comparison of ROC curves between geographic regions or seasons

**Figure 2:**
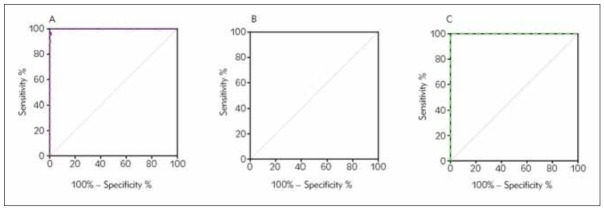
ROC curves of G6PD activity in the prediction of G6PD deficiency of Hefei, Fuyang, and Anqing city. <br>1A Hefei city AUC(95% CI) = 0.999, sensitivity = 100.00%, specificity = 98.92%<br>1B Fuyang city AUC(95% CI) = 1.000, sensitivity = 100.00%, specificity = 99.99%<br>1C Anqing city AUC(95% CI) = 0.999, sensitivity = 100.00%, specificity = 99.87%

We performed ROC analysis to compare the diagnosis efficiency of geographical regions (Figure 2, Table 2). There were no significant differences between HeFei, FuYang, and AnQing in the area under the ROC curves (AUC), as seen in Table 2. Nevertheless, cut-off values determined by the ROC curves are differentiated. The ROC curve analysis revealed a high-predictive value of geographical regions (Table 2). G6PD activity for G6PD deficiency, which in HeFei had AUC (CI 95%) of 0.999, with the optimal cut-off value of 26.55 U/dl (sensitivity, SS: 10.00%; and specificity, SP: 98.92%), in FuYang, having the AUC (CI 95%) of 1.000 with the optimal cut-off value of 6.35 U/dL (SS:100.00%; SP: 99.99%), and in AnQing, had the AUC (CI 95%) of 0.999 with the optimal cut-off value of 7.15 U/dL (SS:100.00%; SP: 99.87%).

**Table 2:** Comparison of ROC curves of G6PD activity in predicting G6PD deficiency of HeFei, FuYang, and AnQing. Abbreviations: HF, HeFei; FY, FuYang; AnQ, AnQing; SP, sensitivity; SP, specificity. a:U/dL

Variables	Cut-offvalues^a^	AUC<br>(95% Cl)	P<br>values	SS (%)	SP (%)	Pairwise comparison of ROC curves		
						Variables	AUC<br>difference	P value
HF	26.55	0.999 (0.998~0.999)	<0.0001	100.00	98.92	HF~FY	0.000974	0.0651
FY	6.35	1.000 (0.999~1.000)	<0.0001	100.00	99.99	FY~AnQ	0.000530	0.2309
AnQ	7.15	0.999 (0.997~1.000)	<0.0001	100.00	99.87	AnQ~HF	0.000443	0.5166

**Figure 3:**
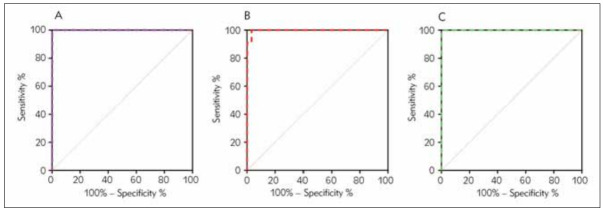
ROC curves of G6PD activity in predicting G6PD deficiency between different seasons. 2A spring AUC (95% CI) = 1.000, sensitivity = 100.00%; specificity = 99.92%, 1B summer AUC(95% CI) = 0.997, sensitivity = 100.00%; specificity = 96.72% and 1C winter AUC(95% CI) = 1.000, sensitivity 100.00%; specificity = 99.98%.

ROC analysis was also conducted on seasons (Figure 3, Table 3). AUC values were not significantly different between each group (as there was no G6PD deficiency confirmed in autumn, values could not be calculated by ROC analysis), except for the significant difference between winter and spring. Accordingly, optimal cut-off values were 21.80 U/dL (SS: 100.00%; SP: 99.92%, AUC (CI 95%)= 1.000), 26.55 U/dL (SS: 100.00%; SP: 96.72%, AUC (CI 95%)= 0.997), 23.16 U/dL (SS:100.00%; SP: 99.98%, AUC (CI 95%)=1.000), of spring, summer, and winter, respectively.

**Table 3:** Comparison of ROC curves of G6PD activity in predicting G6PD deficiency between seasons. Abbreviations: SP, sensitivity; SP, specificity; NA indicates no data available. b:P < 0.05

Variables	Cut-offvalues	AUC<br>(95% Cl)	P<br>values	SS (%)	SP (%)	Pairwise comparison of ROC curves		
						Variables	AUC<br>difference	P value
Spring	21.80	1.000<br>(0.999~1.000)	<0.0001	100.00	99.92	Spring~Summer<br>Spring~Autumn	0.00279NA	0.3467NA
Summer	26.55	0.997<br>(0.995~0.998)	<0.0001	100.00	96.72	Summer~Autumn<br>Summer~Winter	NA 0.00320	NA 0.2799
Autumn	NA	NA	NA	NA	NA	Autumn~Winter	NA	NA
Winter	23.16	1.000<br>(0.999~1.000)	<0.0001	100.00	99.98	Winter~Spring	0.000409	0.0209b

### Comparison of ROC curves between geographic regions in different seasons

**Figure 4:**
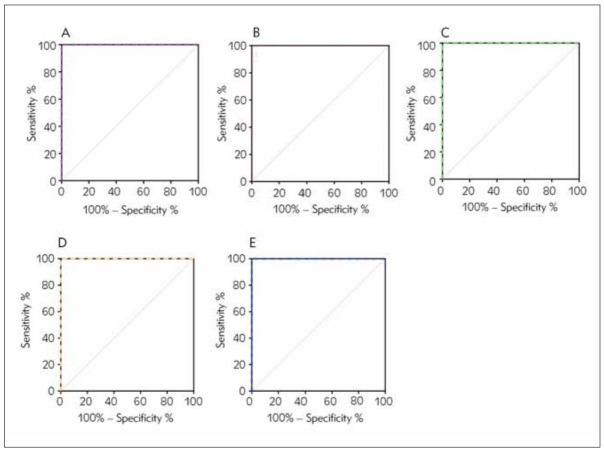
Comparison of ROC curves of G6PD activity in predicting G6PD deficiency between different seasons of different geographic regions<br>4A–4C: for the region of Hefei city; 4A Spring AUC(95% CI) = 1.000, sensitivity = 100.00%, specificity = 99.98% , 4B Summer AUC(95% CI) = 0.997, sensitivity = 100.00%, specificity = 96.69%, and 4C Winter AUC(95% CI) = 1.000, sensitivity = 100.00%, specificity = 99.98%<br>4D for the region of Fuyang city; Summer AUC(95% CI) = 1.000, sensitivity = 100.00%, specificity = 100.00%<br>4E for the region of Anqing city; AUC(95% CI) = 1.000, sensitivity = 100.00%, specificity = 100.00%

To analyze the seasonal characteristics of G6PD activity in different geographical parts, the ROC analysis was performed during different seasons in HeFei, FuYang and AnQing (Table 4, Table 5, Table 6 and Figure 4). Concerning HeFei (Table 4), significant differences between each subgroup were not observed. And based on ROC curve analysis (Table 4), there was a high predictive value for seasons (G6PD activity) when predicting G6PD deficiency, with the AUC (CI 95%) of 1.000, 0.997, 1.000 (Figure 4A, Figure 4B, Figure 4C), and the optimal cut-off values of 21.80 U/dL (sensitivity, SS: 10.00%; and specificity, SP: 99.98%), 26.55 U/dL (SS: 100.00%; SP: 96.69%), 23.16 U/dL (SS: 100.00%; SP: 99.98%), in spring, summer and winter, respectively. As for FuYang and AnQing, sufficient data being not available for between subgroup analysis, we could only obtain the ROC curve (Figure 4D, Figure 4E) characteristics of summer (for FuYang) (Table 5) and winter (for AnQing) (Table 6), and all the AUC (CI 95%) equalled 1.000, with the optimal cut-off values of 6.35 U/dl (sensitivity, SS: 10.00%; and specificity, SP: 100.00%), 7.15 U/dL (SS:100.00%; SP: 100.00%), respectively. However, the p-value of AnQing (subgroup: winter) was 0.0833 > 0.05, and the result of ROC analysis for this category was unreliable.

**Table 4:** Comparison of ROC curves of G6PD activity in predicting G6PD deficiency between different seasons of HeFei. Abbreviations: SP, sensitivity; SP, specificity; NA indicates no data available. b:P < 0.05

Variables	Cut-offvalues	AUC<br>(95% Cl)	P<br>values	SS (%)	SP (%)	Pairwise comparison of ROC curves		
						Variables	AUC<br>difference	P value
Spring	21.80	1.000<br>(0.999~1.000)	<0.0001	100.00	99.98	Spring~Summer<br>Spring~Autumn	0.00383NA	0.2944NA
Summer	26.55	0.997<br>(0.995~0.998)	<0.0001	100.00	96.69	Summer~Autumn<br>Summer~Winter	NA0.00395	NA0.2789
Autumn	NA	NA	NA	NA	NA	Autumn~Winter	NA	NA
Winter	23.16	1.000<br>(0.999~1.000)	<0.0001	100.00	99.98	Winter~Spring	0.000121	0.5053

**Table 5:** Comparison of ROC curves of G6PD activity in predicting G6PD deficiency between different seasons of FuYang. Abbreviations: SP, sensitivity; SP, specificity; NA indicates no data available.

Variables	Cut-offvalues	AUC<br>(95% Cl)	P<br>values	SS (%)	SP (%)	Pairwise comparison of ROC curves		
						Variables	AUC<br>difference	P value
Summer	6.35	1.000<br>(0.976~1.000)	<0.0001	100.00	100.00	Summer~Autumn<br>Summer~Winter	NANA	NANA

**Table 6:** Comparison of ROC curves of G6PD activity in predicting G6PD deficiency between different seasons of AnQing. Abbreviations: SP, specificity; SP, specificity; NA indicates no data available.

Variables	Cut-offvalues	AUC<br>(95% Cl)	P<br>values	SS (%)	SP (%)	Pairwise comparison of ROC curves		
						Variables	AUC<br>difference	P value
Winter	7.15	1.000<br>(1.000~1.000)	0.0833	100.00	100.00	Winter~Spring	0.000418	1.0000

### G6PD activity of G6PD gene mutations and its distribution of mutation frequencies among the geographic regions

**Figure 5:**
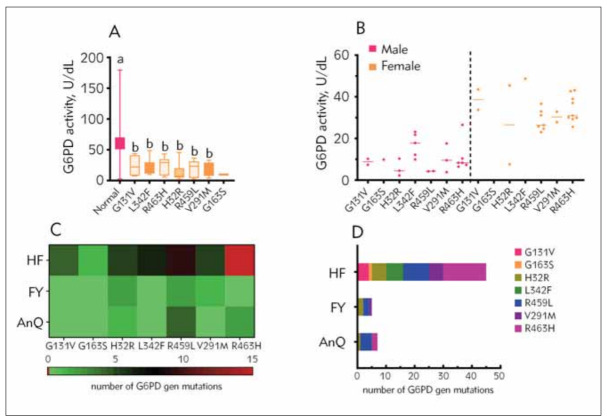
G6PD activity of G6PD gene mutations and its distribution of mutation frequencies among the geographic regions. 5A Comparison of G6PD enzymatic activity of Normal and mutated G6PD enzyme. 5B Comparison of G6PD enzymatic activity between different mutations in Male neonates and Female nenates. 5C Heatmap of mutations in G6PD gene in individuals from different geographic regions. 5D The plot shows the number of G6PD gene mutations in each region. Different G6PD genotypes show significant differences in G6PD activity when boxplots are marked with different letters and nonsignificant differences in G6PD activity when boxplots are marked with the same letters.<br>*Abbreviations*: HF, HeFei; FY, FuYang; AnQ, AnQing.

The phrase »Normal« represented subjects who did not harbour G6PD gene variants. The G6PD activity of the Normal group was significantly higher than all groups of newborns with G6PD gene variation (Figure 5A). Nevertheless, no significant difference was observed for any grouping of G6PD gene variants (Figure 5A). Significant differences existed between males and females regarding G6PD activity, with females having a high G6PD activity (Figure 5B). We also found that G6PD variant R463H was the most frequent (17/57, 29.82%) variation (Figure 5C), followed with R459L (15/57, 26.31%), H32R (8/57, 14.04%), L342F (6/57, 10.53%), V291M (6/57, 10.53%) (Table 3, Figure 5D). Concerning HeFei, R463H was the most commonly mutated allele (15/45, 33.33%), followed by R459L (9/45, 20.00%), L342F (6/45, 13.33%), H32R (5/45, 11.11%), V291M (5/45, 11.11%) and G131V (4/45, 8.89%) (Table 4). Except for H32R, R459L, and R463H (Table 2), no other variants were found in FuYang or AnQing.

## Discussion

In this study, we first reported the prevalence of G6PD deficiency, cut-off values of G6PD activity optimized based on regions and seasons, and the G6PD gene variant spectrum for HeFei, FuYang and AnQing city. It would provide reference data values for preventing and treating G6PD deficiency in Anhui province.

Anhui province has a large population of about 60,000,000; every year, the population is estimated at approximately 400,000 births. For G6PD deficiency, no epidemiological data was previously reported. Our study revealed a prevalence of 0.09%, which is roughly consistent with most provinces in North China and lower than most provinces in South China except Hubei province, neighbouring Anhui province [21]. HeFei, as the capital of Anhui province, located in the middle of Anhui province, had a higher incidence rate (0.10%) than FuYang, located in the north of Anhui province and population numbers being reasonably consistent with HeFei, possessed the incidence rate of 0.04%. But the incidence rate of HeFei was lower than that of AnQing, located along the Yangtze River, which was thought to be associated with the high incidence of G6PD deficiency [22], and had an incidence rate of 0.17%. These results were approximately consistent with the trend of south-highnorth-low. We also found significant differences in age at initial testing and gestational age between the normal and G6PD deficiency groups, which likely happened by chance. A major drawback of this research is that no deficient sample was identified in the female population by the method used to determine G6PD activity. This is indicative because gene analysis indicated the presence of multiple variants in the female population.

We found significant variation in the median results by comparing the G6PD activity obtained for HeFei, FuYang, and AnQing groups, which decreased in the following order: FuYang > AnQing > HeFei. Whereas, uncomprehensively, the data of FuYang and AnQing which obscured the fact was responsible for the conclusion. As presented in Figure 1D, comparing the differences should depend on the spring data rather than the overall data. In fact, the median of G6PD activity was observed in the following order: HF (65.42 (range 59.79–71.25)) > FY (64.10 (range 57.30–71.10)) > AnQ (61.1700 (range 53.37–69.076)). The cause of these discrepancies is poorly defined and remains to be further investigated. Moreover, we revealed that the changing trend of the levels of G6PD activity was consistent with that first decreasing and then increasing with the season, followed the order of winter (71.9 range (66.44–77.65)) > spring (65.42 range (59.74–71.2375)) >autumn (48.89 range (43.48–54.79)) > summer (45.65 range (37.91–52.47)). One plausible mechanism is that temperature may affect the activity of G6PD. High temperatures could decrease the activity of G6PD in summer, while low temperatures increase it in winter.

We have established cut-off values predicated on geographic regions and seasons and demonstrated that cut-off values varied with different geographic regions and seasons. The optimal derived cut-offs for spring, summer and winter to detect G6PD deficiency in the region of HeFei, were 21.80 U/dl (SS:100.00%; SP: 99.92%, AUC(CI 95%)= 1.000), 26.55 U/dl (SS: 100.00%; SP: 96.72%, AUC (CI 95%)= 0.997) and 23.16 U/dl (SS: 100.00%; SP: 99.98%, AUC (CI 95%)= 1.000), respectively, which agreed with the overall results (Table 3). Additionally, the best cut-off values of FuYang and AnQing differed from those of HeFei, which was also supported by the result of the ROC curve, and the cut-off values of FuYang and AnQing were 6.35 (sensitivity 100%, specificity 99.99%) and 7.15 (sensitivity 100%, specificity 99.87%), respectively.

In our research, the results were considered the most suitable cut-off values for FuYang and AnQing because there was insufficient data to establish different cut-off values constructed on seasons. To our knowledge, no previous study reported accurate cutoff values of Anhui province relying on seasonal factors. It was filled in this research, and before this study, the cut-off values were set to 35 U/dl, followed by the manufacturer's instructions, which was significantly higher than the result of our research. Consequently, our results provided a precise approach to facilitate G6PD deficiency screening, achieving higher screening accuracy, reducing unnecessary anxiety resulting from false positives, and saving medical resources.

We found that G6PD gene variant R463H was a significant source of hotspot mutations in HeFei and identified the gene mutated frequency arranged in the order of R459L (24.56%) > H32R (14.04%) > L342F (10.53%) > V291M (10.53%), of which overlapped with the gene frequency of Chongqing and Guangxi [21]. R463H emerged in the past 5000 to 6000 years [23], and was considered to have a significantly higher bilirubin level than that of R459L among hyperbilirubinemia neonates with G6PD deficiency [24]. Moreover, R459L is thought to have commenced in the past 3125 to 3750 years [24], located at 12 bases from R463H. Those two sites are located in the conserved regions, which contain more than 10 highly amino acids, and more importantly, these two variants stay closer to the first binding site of NADP (386-387) in space. Consequently, the potential mechanisms underlying the effect of the binding process between the G6PD enzyme and NADP [25] may influence the G6PD activity. Our research results likely indicated that individuals, especially males carrying R463H and R459L, had to attach greater importance and higher priority to manage.

There remain several shortcomings in this study. Firstly, due to the limited investigation and the lack of diagnosis data from autumn, the G6PD activity cutoff values have not been established for autumn. Likewise, relying on different seasons, we could not establish the best suitable cut-off values of G6PD activity for FuYang and AnQing city. Thirdly, a small proportion of G6PD deficiency cases may be missed because we evaluated the positive subjects relying exclusively on hotspot mutations of the G6PD gene, which accounts for approximately 95% of causative reasons for G6PD deficiency individuals in the Chinese population, as the previous study reported [21]. Actually, there are continuous reports describing additional findings in novel mutations of the G6PD gene [26] [27], which is essential for identifying G6PD deficient patients. With the development of gene sequencing, high throughput, low-cost assay for detecting the mutations of the G6PD gene may soon become a reality. Next Generation Sequencing (NGS) is rapidly becoming an essential diagnostic tool for genetic disease patients [28] [29]. The advantage of NGS, in contrast, is that we can direct sequence the 13 exons of the flanking sequences of the G6PD gene other than G6PD hotspot mutations.

In addition, we have first investigated the prevalence, cut-off values and variant spectrum of G6PD deficiency patients, depending on a population cohort of 31482 neonates from Newborn Screening among HeFei, FuYang, and AnQing. Our study will provide data to promote G6PD deficiency screening being integrated into the Newborn Screening program in Anhui province for earlier identification of neonates at risk of G6PD deficiency.

## Dodatak

### Authors contributions

The study was conceived and designed by all authors. Hui Li is the study leader. Yah Ch’ih conducted the genetic test for G6PD deficiency. Meiling Li, Yulei Luo and Hao Liu performed the G6PD newborn screening test. Junyang Xu conducted the quality control of the newborn screening test. Qingqing Ma and Wangsheng Song were responsible for diagnosing and treating positive subjects. Hui Li gathered the newborn screening data and performed the statistical analysis of experimental data. Ziyu Shao undertook the project coordination. Yah Ch’ih wrote and revised the manuscript. All authors have read and supported the final draft.

### Funding

This work was funded by the HeFei Municipal Health Commission (#2019-20).

### Conflict of interest statement

All the authors declare that they have no conflict of interest in this work.
